# Meibomian Gland Infarction of the Eyelid Mimicking a Tumor: A Case Report

**DOI:** 10.7759/cureus.98404

**Published:** 2025-12-03

**Authors:** Daisuke Hasegawa, Yui Nishijima, Satoko Kujiraoka, Naoyuki Matsumoto, Tatsuya Mimura

**Affiliations:** 1 Department of Ophthalmology, Tsurumi University School of Dental Medicine, Yokohama, JPN; 2 Department of Diagnostic Pathology, Tsurumi University Dental Hospital, Yokohama, JPN; 3 Department of Pathology, Tsurumi University School of Dental Medicine, Yokohama, JPN; 4 Department of Ophthalmology, Teikyo University School of Medicine, Tokyo, JPN; 5 Department of Ophthalmology, Tsurumi University Dental Hospital, Yokohama, JPN

**Keywords:** eyelid mass, eyelid tumor mimic, histopathology, keratinized lesion, meibomian gland infarction

## Abstract

This report describes a rare case of a keratinized eyelid mass associated with meibomian gland infarction arising from the lower eyelid. An 84-year-old man presented with a three-month history of swelling along the medial margin of the left lower eyelid, which had rapidly enlarged and become tender during the two weeks prior to presentation. He had no history of ocular surgery, and examination revealed a firm, elastic mass that was surgically excised in the outpatient operating room. Histopathological evaluation showed no evidence of malignancy; rather, the lesion was covered by stratified squamous epithelium with melanin pigmentation, beneath which markedly dilated meibomian gland ducts were observed. The stroma contained abundant sebaceous material and keratinous debris, findings consistent with a keratinized lesion secondary to meibomian gland infarction. Because meibomian gland infarction can clinically mimic malignant eyelid tumors or chalazia, careful differential diagnosis is required. This case highlights an uncommon presentation characterized by rapid enlargement and distinctive keratinized pathology.

## Introduction

Eyelid tumors encompass a wide spectrum of etiologies, ranging from benign chalazia to malignant sebaceous carcinoma, making accurate differentiation essential. In elderly patients in particular, tumors and functional abnormalities of the meibomian glands are of clinical relevance. The meibomian glands are holocrine sebaceous glands distributed within the upper and lower tarsal plates; their secretions (meibum) form the lipid layer of the tear film and play a critical role in preventing evaporation and maintaining tear film stability [1]. When evaluating eyelid masses, the differential diagnosis must therefore include a broad range of benign and malignant conditions. Meibomian gland-related abnormalities are clinically and histologically diverse, and in older individuals, glandular dysfunction or structural changes may contribute to mass formation [1].

Meibomian gland dysfunction (MGD), characterized by ductal obstruction and alterations in the quality or quantity of meibum, is a major cause of evaporative dry eye disease [1]. Recent studies highlight the central role of hyperkeratinization of the ductal epithelium in the onset and progression of MGD. Dermatologic perspectives have proposed that excessive keratin accumulation at the orifices contributes to obstruction and disease development [2]. Emerging evidence also implicates the impaired regenerative capacity of acinar epithelial cells in the pathogenesis of MGD. Hyperkeratinization-induced obstruction may initiate a vicious cycle of intraductal stasis, acinar atrophy, and functional decline [3]. Chronic MGD is additionally associated with inflammation (“meibomitis”), in which stagnant meibum can promote bacterial proliferation and inflammatory responses [4].

Histopathologic studies have demonstrated that even mild or “non-obvious” MGD can exhibit hyperkeratinization or keratinous plugs (keratotic clusters), which may not be readily detected through conventional external examination [5]. The International Workshop on Meibomian Gland Dysfunction defines MGD as a chronic condition involving terminal duct obstruction and/or qualitative or quantitative changes in glandular secretions, which subsequently lead to tear film instability and ocular surface disease [6]. Japanese clinical guidelines also describe the main ducts as being lined by stratified squamous epithelium, positioning hyperkeratinization as a central pathological mechanism [7].

In contrast, cases in which keratinous material accumulates extensively due to ductal obstruction or degradation of stagnant meibum - forming a discrete mass - are exceedingly rare. Such lesions, termed meibomian gland infarction or concretion, may present with rapid enlargement and tenderness, potentially mimicking malignant tumors. However, reports of this entity in the literature remain limited.

In this report, we present a rare case of a rapidly enlarging, tender lower eyelid mass in an elderly man, which was clinically suspicious for malignancy but was ultimately diagnosed as a benign keratinized lesion associated with meibomian gland infarction based on histopathological findings.

## Case presentation

**Figure 1:**
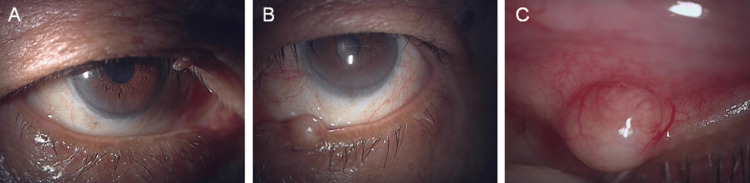
Clinical photographs of the right (A) and left (B) eyes of an 84-year-old man, with a close-up view of the keratinized mass located on the medial aspect of the left lower eyelid (C).

An 84-year-old man noticed a small nodular swelling on the medial aspect of the left lower eyelid margin approximately three months earlier. He also reported a mild foreign body sensation and dryness in the left eye during this period. Two weeks before presentation, the lesion began to enlarge rapidly and became tender, prompting consultation at our department. At the initial examination, the lesion appeared as a smooth, dome-shaped nodule without telangiectasia, ulceration, or madarosis, and no features suggestive of malignancy were observed. At the initial visit, best-corrected visual acuity was 20/15 (× +3.25 = -3.0 Ax90) in the right eye and 20/20 (× +3.5 = -2.5 Ax90) in the left eye. Intraocular pressure was 16 mmHg in both eyes. Anterior segment examination revealed mildly shallow anterior chambers and bilateral cataracts, predominantly characterized by nuclear sclerosis (Figure 1). His systemic history was unremarkable, with no internal medical conditions or prior ophthalmic surgeries.

The left lower eyelid lesion was located near the eyelid margin and was palpable as a firm, poorly mobile, elastic mass (Figure 1). The overlying skin showed neither erythema nor erosion, and no regional lymphadenopathy was detected. In addition, the lesion had shown rapid enlargement over the preceding two weeks, and fine telangiectatic vessels were observed on its surface, raising concern for a potentially malignant process. Because the differential diagnosis included malignant eyelid tumors as well as chalazion, surgical excision was planned in the outpatient operating suite.

Under local infiltration anesthesia, excision of the mass was performed. A capsulated lesion was initially suspected, and we planned to incise the capsule and curette the internal contents. However, the lesion was firmly adherent to the surrounding tissues and uniformly hard, making separation difficult. The capsule and mass were tightly fused. Therefore, the lesion was detached at its base using a sharp blade and removed en bloc. Intraoperative bleeding was minimal and was easily controlled with one minute of gauze compression. Ofloxacin ophthalmic ointment was applied to the excision site, which was then covered with sterile gauze secured with tape for infection prophylaxis.

**Figure 2:**
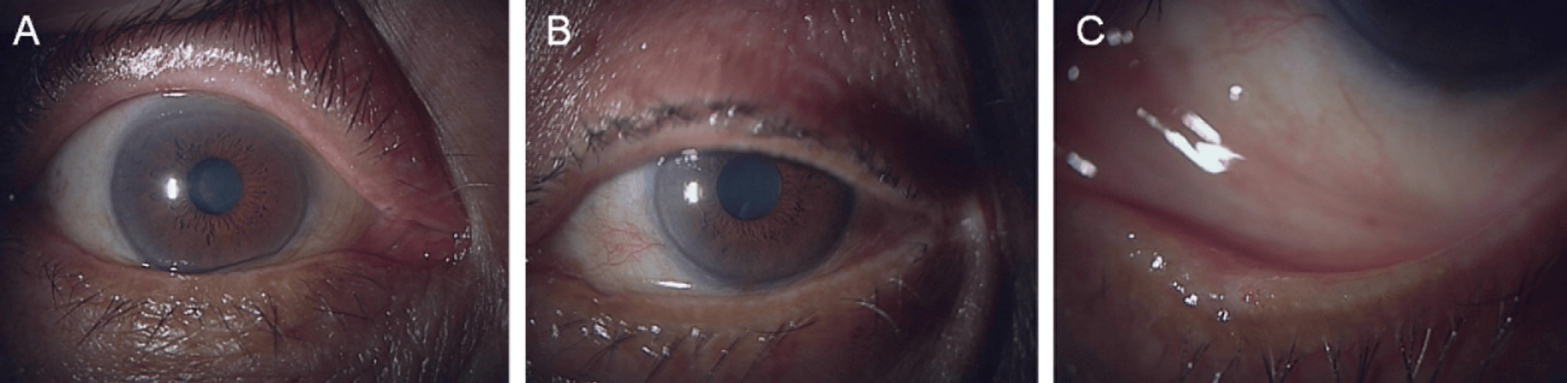
Postoperative eyelid findings of the right (A) and left (B) eyes, one week after excision of the left lower eyelid mass. The surgical site demonstrates good epithelialization, without evidence of inflammation (C).

At the one-week postoperative follow-up, the wound was well epithelialized, with no signs of inflammation, such as erythema or swelling (Figure 2).

**Figure 3:**
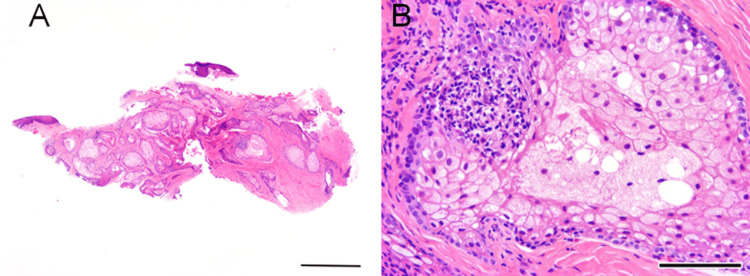
Histopathological findings of the excised left lower eyelid mass (hematoxylin-eosin stain). (A) Left: low-magnification view (scale bar = 1 mm). (B) Right: high-magnification view (scale bar = 50 µm).

Histopathological examination of the excised specimen revealed no evidence of malignancy. The lesion was lined by stratified squamous epithelium with melanin deposition, and dilated meibomian gland ducts were identified beneath the epithelial layer. The stroma contained abundant sebaceous material and keratinous debris. Based on these findings, the lesion was diagnosed as a keratinized mass associated with meibomian gland infarction (obstruction) (Figure 3).

## Discussion

This case presented as a rapidly enlarging, tender mass in the left lower eyelid, raising clinical suspicion for a malignant tumor such as sebaceous carcinoma. Several conditions may exhibit similar features, including chalazion, lipoma, and meibomian gland carcinoma. The firm consistency of the mass and its recent rapid growth heightened concern for these potential mimickers. However, histopathological examination revealed a benign keratinized lesion associated with meibomian gland infarction/concretion. Such presentations are extremely rare and provide valuable insights from both clinical and pathological perspectives.

First, it is important to consider the spectrum of meibomian gland disorders. MGD is a multifactorial condition, characterized by terminal duct obstruction, altered quality or quantity of secretions, and chronic inflammation. Recent reviews have emphasized that MGD is not merely the result of lipid stasis, but involves ductal abnormalities and structural remodeling, often associated with hyperkeratinization. Hyperkeratinization of the terminal ducts can lead to orifice obstruction, resulting in stasis, inflammation, and eventual glandular atrophy [8]. In animal models, such as rabbits, ductal obstruction induces hyperkeratinization, granulation tissue reaction, and cystic dilatation, demonstrating significant morphological changes [9].

In our case, histopathology confirmed ductal dilatation and the presence of keratinous material, consistent with these pathological models. Notably, the accumulation of keratin associated with infarction/concretion formed a mass, which manifested clinically as rapid enlargement and tenderness, representing a macroscopic manifestation of MGD progression.

From a differential diagnostic standpoint, it was reasonable to consider a malignant tumor, particularly sebaceous carcinoma. Sebaceous carcinoma can closely mimic chalazion or other benign eyelid lesions, often leading to delayed diagnosis [10]. Reports also describe cases of sebaceous carcinoma presenting as large, chalazion-like lesions [11], highlighting the difficulty of distinguishing benign from malignant lesions based solely on clinical findings.

Comparison with other drug- or inflammation-related meibomian gland disorders is also relevant. Immune checkpoint inhibitors, such as pembrolizumab, have been reported to induce immune-related MGD with surface atrophy and deformity [12]. Similarly, cancer therapies, including trastuzumab, pertuzumab, and anastrozole, have been associated with abnormal lipid deposition and chronic inflammatory changes in the meibomian glands and conjunctiva [13]. While these represent distinct pathogenic mechanisms, they underscore the potential for structural changes in the gland.

From a treatment perspective, this case demonstrated favorable postoperative healing, with minimal risk of inflammation recurrence and no functional sequelae. Surgical excision appears to be a relatively safe and effective intervention for benign, infarction-related lesions. However, alternative management strategies, such as warm compresses, meibomian gland probing, or periodic monitoring, may be useful in cases where excision is challenging.

This case report has several limitations. First, as a single case, it cannot be generalized to all keratinized lesions associated with meibomian gland infarction/concretion. Specifically, the extent to which rapid enlargement and tenderness are characteristic features remains unclear and requires the accumulation of additional cases. Second, the precise temporal course of lesion development and the predisposing events leading to ductal obstruction and keratin accumulation were not directly observed, limiting conclusions about whether this lesion represents part of the natural progression of MGD or a distinct pathological entity. Third, no adjunctive assessments, such as meibography, detailed lipid analysis, or inflammatory marker evaluation, were performed, precluding comprehensive evaluation of glandular dysfunction in relation to mass formation. These limitations necessitate cautious interpretation, and future case reports may further elucidate the pathophysiology of this rare entity.

## Conclusions

We report an extremely rare case of a rapidly enlarging, tender eyelid mass that was clinically suggestive of malignancy, but histopathologically diagnosed as a benign keratinized lesion associated with meibomian gland infarction. The pathological findings of ductal dilatation and keratin accumulation align with hyperkeratinization and obstruction mechanisms observed in MGD, illustrating the complexity and diversity of meibomian gland disorders. This case underscores the importance of considering infarction-related meibomian gland lesions in the differential diagnosis of eyelid masses and highlights the critical role of histopathological evaluation in avoiding misdiagnosis of malignancy. Accumulation of similar cases will help clarify the clinical characteristics and etiology of this rare pathological entity.
